# Roles and mechanism of IL-11 in vascular diseases

**DOI:** 10.3389/fcvm.2023.1171697

**Published:** 2023-05-26

**Authors:** Jiacheng Wu, Wenrui Ma, Zhihua Qiu, Zihua Zhou

**Affiliations:** ^1^ Department of Cardiology, Union Hospital, Tongji Medical College, Huazhong University of Science and Technology, Wuhan, China; ^2^ Hubei Key Laboratory of Biological Targeted Therapy, Union Hospital, Tongji Medical College, Huazhong University of Science and Technology, Wuhan, China; ^3^ Hubei Engineering Research Center for Immunological Diagnosis and Therapy of Cardiovascular Diseases, Union Hospital, Tongji Medical College, Huazhong University of Science and Technology, Wuhan, China; ^4^ Department of Radiology, Guangdong Provincial People’s Hospital (Guangdong Academy of Medical Sciences), Southern Medical University, Wuhan, China

**Keywords:** vascular diseases, IL-11, ERK, STAT3, vascular smooth muscle cells

## Abstract

Vascular diseases are the leading cause of morbidity and mortality worldwide. Therefore, effective treatment strategies that can reduce the risk of vascular diseases are urgently needed. The relationship between Interleukin-11 (IL-11) and development of vascular diseases has gained increasing attention. IL-11, a target for therapeutic research, was initially thought to participate in stimulating platelet production. Additional research concluded that IL-11 is effective in treating several vascular diseases. However, the function and mechanism of IL-11 in these diseases remain unknown. This review summarizes IL-11 expression, function, and signal transduction mechanism. This study also focuses on the role of IL-11 in coronary artery disease, hypertension, pulmonary hypertension, cerebrovascular disease, aortic disease, and other vascular diseases and its potential as a therapeutic target. Consequently, this study provides new insight into the clinical diagnosis and treatment of vascular diseases.

## 1. Introduction

Vascular diseases, including hypertension, coronary artery disease, aortic dissection, and peripheral artery disease, are the leading cause of morbidity and mortality worldwide (1). In 2019, 1.28 billion people aged 30–79 had high blood pressure (2), and deaths from coronary artery disease are estimated to be 8.9 million annually (3). Aging increases the risk of developing vascular diseases. Unfortunately, no effective treatment for vascular diseases has been developed yet. Thus, novel therapeutic approaches for treating or managing vascular diseases are urgently needed.

IL-11 is a member of the IL-6 cytokine family and directly impacts vascular function similar to other family members, including IL-6, leukemia inhibitory factor, oncostatin M, cardiac trophin 1, and IL-27 (1). Previous studies have found that IL-11 enhances megakaryocyte progenitor maturation in the bone marrow *in vivo* and platelet production (4). Recombinant human IL-11 (rhIL-11) treats chemotherapy-associated thrombocytopenia (5). More recently, the roles for IL-11 in promoting the progression of colorectal (6), breast (7) and gastric (8) have emerged. Additionally, IL-11 has been implicated in the pathogenesis of inflammatory diseases. IL-11 has been shown to promote fibrosis of the heart (9), kidney (10), lung (11), and liver (12). In healthy individuals, IL-11 expression in plasma is nearly undetectable. It has been observed that under pathological conditions such as inflammation and fibrosis, the expression of IL-11 is significantly altered through the stimulation of TGF-β and IL-1. Several studies have shown that IL-11 participates in the occurrence and progression of cardiovascular diseases. This review evaluated the function and mechanism of IL-11 in vascular wall cells and vascular diseases. In addition, the prospect of using IL-11 in diagnosing and treating vascular diseases was explored.

## 2. IL-11 signaling

The human IL-11 genome sequence is 7 kb in length and consists of five exons and four introns. The IL-11 gene is mapped to the long arm of human chromosome 19 (19q13.3–q13.4) by *in situ* hybridization (13). The IL-11 precursor protein is 199 amino acids in length, and its N-terminal signal peptide with 21 amino acid residues is cleaved before cell release, resulting in the formation of the mature secreted protein consisting of 178 amino acids with a molecular weight of roughly 20 kDa-IL-11 (14, 15). IL-11, a member of the IL-6 family of cytokines, is the closest cytokine to IL-6. Structurally, IL-11 has additional helical extension elements around its four helical bundles and shares the same four helical bundles as IL-6. These helical extension elements are crucial for IL-11's attachment to its receptor (16).

GP130 receptors is a ubiquitously expressed glycosylated type I membrane protein (17). The expression of IL-11Rα is cell-type specific, including fibroblasts (18), smooth muscle cells (19) and endothelial cells (20). The classical signal transduction mechanism of IL-11 involves the interaction of a soluble form of IL-11 with IL-11RA and GP130 receptors in the same cell to create a trimeric complex. Two identical trimeric complexes ultimately combine, forming a functional hexamer complex (17, 21). The hexamer complex exerts pathophysiological functions via three vital signaling pathways. (1) JAK/STAT3 signaling pathway: The recruited JAK mediates the phosphorylation of GP130 tyrosine residues, thus phosphorylating and activating STAT3. Two phosphorylated STAT3 polymerize and enter the nucleus to regulate gene transcription, affecting cell proliferation and migration (22). (2) RAS/ERK pathway: By combining with SHP2 (a tyrosine phosphatase), phosphorylated tyrosine at the proximal end of the GP130 membrane activates the RAS/ERK pathway, which results in vascular inflammation and fibrosis (23, 24). (3) PI3K/mTOR pathway: GP130 activates the PI3K-AKT-mTOR pathway without tyrosine phosphorylation, promoting the migration and angiogenesis of cancer cells (23); however, no specific role of this pathway in IL-11 has been reported.

In addition, IL-11 may participate in prime signal transduction in two other non-classical ways: (1) Trans signal transduction: the extracellular part of IL-11RA, cleaved by integrin metalloproteinase 10, forms a soluble receptor and binds to IL-11. The IL-11 combines with cells that express GP130 but not IL-11RA, which mediates signal transduction (25). (2) Cluster signal transduction: IL-11RA-expressing cells can bind to IL-11, termed transmitter cells.These transmitter cells interact with adjacent receiver cells expressing membrane bound GP130, to triggers signal transduction (26).. Whether these transductions account for the downstream pathway via the hexamer complex, such as classical IL-11 signal transduction, remains to be confirmed (27).

## 3. Expression and role of IL-11 in vascular wall cells

**Figure 1:**
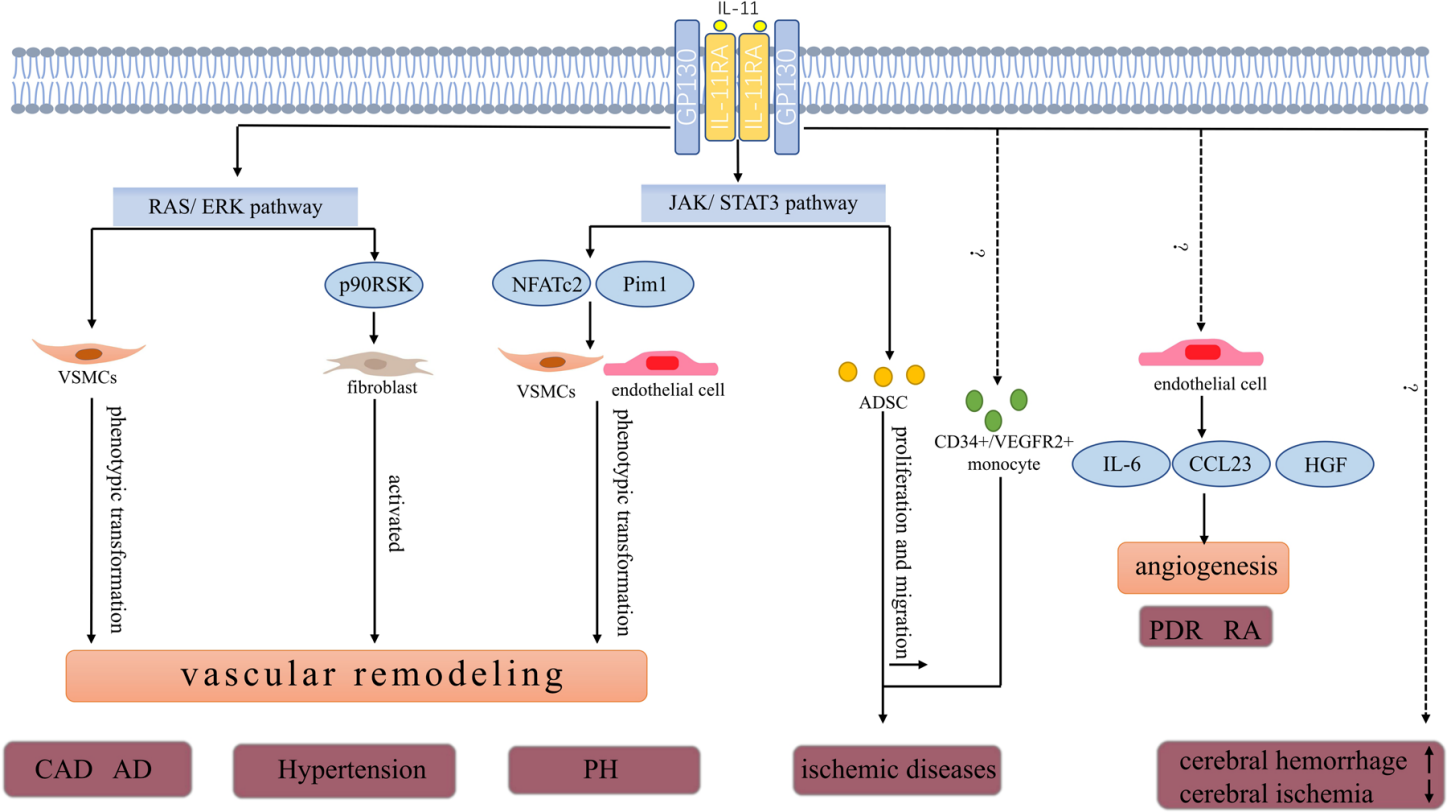
Pathophysiologic roles of IL-11 in vascular diseases. IL-11 is involved in the progression of CAD. High IL-11 expression may increase the risk of developing restenosis after stent implantation. In a model of carotid artery plaque formation, IL-11 induces phenotype transformation of VSMCs by activating the ERK signal and promotes vascular fibrosis and plaque formation. AD is a fatal vascular disease the level of IL-11 in plasma and thoracic aorta with acute thoracic aortic dissection increased. IL-11 potentially stimulates phenotype transformation of VSMCs in an ERK-dependent manner. The phenotype transformation of VSMCs further promotes aortic remodeling and increased risk of aortic dissection. IL-11 promotes the development of hypertension and may also be associated with the ERK pathway. As discussed in the text, the expression of IL-11 and IL-11RA was upregulated in the pulmonary artery and serum of patients with PH. The JAK/STAT3 pathway, a key downstream pathway of IL-11, promotes pulmonary vascular remodeling by increasing the expression of Pim1 (a proto-oncogene serine/threonine protein kinase) and NFATc2 in endothelial and VSMCs. IL-11 level was positively correlated with the risk of developing cerebral hemorrhage and negatively correlated with the severity of cerebral ischemia.In ischemic diseases,IL-11 increases the number of circulating CD34+/VEGFR2+ monocytes, which ultimately improves blood supply. In addition, ADSCs promote angiogenesis in ischemic tissues. IL-11 enhances ADSCs’ proliferation and migration through the STAT3 signaling pathway to increase blood perfusion. In PDR and RA, IL-11 is involved in angiogenesis by inducing the secretion of HGF, CCL23, and IL-6 in HUVECs.CAD, coronary artery disease; AD, aortic dissection; PH, pulmonary hypertension; PDR, proliferative diabetic retinopathy; RA, rheumatoid arthritis; ADSCs, adipose-derived mesenchymal stem cells; VSMCs, vascular smooth muscle cells; NFATc2, nuclear factor of activated T cells 2; CCL23, C-C motif chemokine ligand 23; HGF, hepatocyte growth factor.

The vascular wall acts as a physical barrier for blood cells and is also considered a functional endocrine organ. Different cytokines can regulate the function of vascular wall cells (1). Endothelial cells, smooth muscle cells, and fibroblasts in the vascular wall of healthy humans have low interleukin (IL)-11 levels. Nevertheless, the expression of IL-11 in vascular wall cells fluctuates in response to the vascular inflammation, fibrosis, and irritation of IL-1 and TGF-β (Table 1) (28, 29). IL-11 promotes the pathological process of different vascular diseases by impairing the function of vascular wall cells (Figure 1) (30–32).

**Table 1:** Expression of IL-11 in various cells and its potential functions.

Tissue/Cell (human/mouse)	Expression of IL-11/IL-11RA	Model/Disease	Effect of IL-11	Refs.
EC				
HUVECs	IL-11 ↑	CMV stimulation		(17)
HUVECs		CTL damage	Resist HUVECs damage	(25)
HUVEC, HPMEC		H_2_O_2_ damage	Decrease cell death	(26)
Human RA ST endothelial cells	IL-11 ↑	RA	Induce angiogenesis	(38)
Human retinal microvessels Endothelial cells	IL-11, IL-11RA ↑	PDR patients	Induce angiogenesis	(64)
Mouse endothelial cells		Human skin allograft model	Protect endothelial cells by survivin	(19)
VSMCs				
Human aorta VSMCs	IL-11 ↑	TGF-β stimulation		(31)
Mouse aorta VSMCs	IL-11 ↑	Aortic coarctation model Ang II stimulation	Induce VSMCs phenotypic transformation	(32)
Human coronary arteries VSMCs	IL-11 ↓	Early coronary atherosclerosis patients		(18)
PASMCs	IL-11, IL-11RA ↑	IPF and PH patients	Induce PASMCs phenotypic transformation	(51)
Mouse aorta VSMCs	IL-11 ↑	Marfan syndrome model	IL-11 inhibition reduces aortic inflammation, fibrosis	(13)
Mouse carotid artery VSMCs		Carotid artery injury + high fat diet model	IL-11 inhibition reduces VSMCs phenotypic transformation and vascular fibrosis	(20)
Fibroblast				
Human lung Fibroblasts	IL-11 ↑	IPF patients	Induce fibroblasts transform into myofibroblasts	(51)
Mouse thoracic aorta fibroblasts	IL-11 ↑	AngII hypertension model	IL-11 inhibition reduces vascular fibrosis	(21)
Serum				
Human venous blood	IL-11 ↓	CAD patients		(42)
Human arterial blood	IL-11 ↑	CAD patients		(41)
Human serum	IL-11 ↑	Spontaneous intracerebral hemorrhage	Associate with hydrocephalus and mortality after intracerebral hemorrhage	(55)
Human serum	IL-11 ↑	HICH	Associate with disease severity and prognosis	(56)
Human serum	IL-11 ↑	AD patients		(60)
Mouse serum	IL-11 ↑	PE		(49)

### 3.1. Endothelial cells

Vascular endothelial cells, located in the innermost lining of the vascular wall, modulate vascular tension, vascular activity, immune function, etc. Endothelial dysfunction is closely associated with vascular disease (33). In endothelial cells, IL-11 is poorly expressed physiologically. Nonetheless, under endothelial cell damage caused by tumor infection and other events, the expression of IL-11 or IL-11RA is altered. Human cytomegalovirus has strongly driven the upregulation of the IL-11 protein in human umbilical vein endothelial cells (HUVECs) (28). Meanwhile, the vascular endothelial growth factor promotes the overexpression of the IL-11 gene in HUVECs (34).

IL-11 protects endothelial cells *in vitro*. In HUVECs, pretreatment of rhIL-11 partially resists HUVECs damage initiated by cytotoxic T lymphocytes (35). Similarly, rhIL-11 decreases HUVECs and human pulmonary microvascular endothelial cell death evoked by H_2_O_2_ (36). Survivin, an antiapoptotic protein, is rarely expressed in adult individuals but can be re-expressed in endothelial cells during angiogenesis to protect endothelial cells (37). IL-11 enhances the expression of survivin in HUVECs to resist endothelial cell apoptosis via the STAT3-dependent pathway (38).

Animal experiments have shown that IL-11 mediates the effect of angiogenesis. In immunodeficiency mice models with human skin transplants, intradermal injection of rhIL-11 (500 ng/day) prevented microvascular loss of the transplanted skin by mediating the expression of survivin (30). Further proteomic analysis revealed that HUVECs secretes abundant C-C motif chemokine ligand 23 (CCL23), hepatocyte growth factor (HGF), and IL-6. The secretion of the three proteins is induced by IL-11, HGF, CCL23, and IL-6, which participates in angiogenesis (39). These results imply that IL-11 potentially inhibits apoptosis and angiogenesis of endothelial cells.

### 3.2. Vascular smooth muscle cells

Vascular smooth muscle cells (VSMCs) are specialized cells in the middle layer of blood vessels, known to regulate vascular tension and blood pressure. Under normal physiological conditions, VSMCs proliferate at a slow rate and over-express contractile proteins. Pathological arterial traumas, including hypertension, atherosclerosis, and aortic remodeling, cause a phenotypic switch of VSMCs from a contraction phenotype to a synthesis phenotype characterized by proliferation, migration, and secretion of extracellular matrix. In pathological vascular injuries, including hypertension, atherosclerosis, and aortic remodeling, VSMCs exhibit a phenotypic transformation from contraction phenotype to synthesis phenotype (40). Typically, IL-11 is poorly expressed in VSMCs. Interestingly, IL-1 activates the production of IL-11 in a dose-dependent manner (29, 41). Additionally, IL-11 levels in human aortic VSMCs are consistently and substantially elevated by TGF-β (42).

Early research demonstrated that rhIL-11 partially reverses the activation of NF-kB and the secretion of its downstream IL-8 and IL-6 cytokines, prompted by bFGF in human VSMCs *in vitro*, thereby inhibiting the proliferation of VSMCs (43). However, recent studies have confirmed that IL-11 induces phenotype transformation of VSMCs by activating the ERK signal of VSMCs. Inhibition of the IL-11 signal decreases the phenotypic transformation of VSMCs initiated by TGF-β and Ang Ⅱ (42). Notably, In vivo models revealed that the deposition of perivascular collagen increases and the vascular wall of heart tissue thickens in smooth muscle specific IL-11 transgenic mice (44). These results indicate that IL-11 can trigger the phenotypic transformation of VSMCs and participate in vascular remodeling. In another research, the right carotid artery of ApoE mice on a high-fat diet was injured using a guide wire to establish a vascular restenosis model after stent implantation. Injection with X203 (IL-11 neutralizing antibody) reduced the number of VSMCs, matrix metalloproteinase 2 (MMP2) level, and collagen content. Meanwhile, treatment with X203 was linked to a substantial rise in smooth muscle protein 22α positive cells in vascular plaques, indicating that X203 potentially maintain the contractile phenotype of VSMCs and shorten the phenotypic transformation of VSMCs. X203 also lowered collagen content in the undamaged carotid artery and improved the arterial remodeling induced by hyperlipidemia in ApoE mice fed with high fat diet al.one (31). Therefore, although early studies believed that IL-11 inhibits the proliferation of VSMCs, a current study demonstrated that IL-11 is more likely to promote the phenotypic transformation of VSMCSs, resulting in vascular remodeling. This contradictory result may be attributed to different disease models or provocative methods (31).

### 3.3. Fibroblasts

Fibroblasts are the main cell types that constitute the adventitia of blood vessels, and regulates the response of blood vessels to stress or injury (32). In a typical vascular wall, fibroblasts mainly secrete extracellular matrix, preserving the structure of the vascular wall. Low IL-11 protein and mRNA levels are physiologically detectable in fibroblasts. Due to environmental stimulus, fibroblasts become active and participate in vascular remodeling (45, 46). A previous study revealed that IL-11 secretion in fibroblasts is induced by TGF-β in a time and dose dependent manner (29).

IL-11 contributes to the activation of fibroblasts, triggering perivascular fibrosis and inflammation (47). In rheumatoid arthritis, IL-11 promotes the migration of fibroblasts to the joints and the secretion of cytokines (18). AngⅡ drives bimodal activation of ERK1/2 in primary cultured rabbit thoracic aortic adventitia fibroblasts. The first activation of ERK1/2 is prompted by AngⅡ, whereas the over-expression of IL-11 mediates the second activation. p90RSK is a direct downstream effector of ERK1/2 signal transduction. IL-11 potentially drives the release of ACTA2 and collagen type I 1 chain protein in fibroblasts, inducing vascular inflammation, fibrosis, and remodeling of the vascular adventitia by activating p90RSK through the non-classical ERK pathway (32).

## 4. The relationship between IL-11 and vascular diseases

### 4.1. Coronary artery disease

Coronary artery disease (CAD) includes all disorders with limited myocardial blood flow. Plaque formation caused by coronary atherosclerosis is the primary factor of blood flow restriction. Unstable plaque rupture and thrombosis may cause acute coronary syndrome (48). Research has shown that inflammatory cytokines and growth factors, including TGF-β, IFN-γ, TNF-α, IL-1, and IL-6, are involved in the progression of CAD (49). The transcription of mRNA and the subsequent expression of IL-11 are downregulated in coronary artery tissues and venous blood of CAD patients (29, 50). Conversely, a previous study reported that IL-11 expression was higher in the arterial blood of CAD patients than in non-CAD patients (29, 50, 51). These conflicting outcomes could be a consequence of IL-11 differential expression at various phases of CAD. As a result, IL-11 potentially serves several purposes in the evolution of CAD.

To determine the role of IL-11 in CAD, Obana M et al. established a myocardial infarction model by ligating the left coronary artery of mice. Intravenous infusion of rhIL-11 for four consecutive days increased the micro blood supply and reduced the death of myocardial cells at the edge of the infarct area. Furthermore, the rhIL-11 reduced the fibrotic size 14 days after myocardial infarction and improved cardiac function. In addition, compared with the wild-type mice, the fibrosis of heart-specific STAT3 deletion mice was aggravated (52). IL-11 also enhances myocardial ischemia-reperfusion injury. In the rat model of heart ischemia-reperfusion, intravenous injection of rhIL-11 reduces cardiomyocyte apoptosis and improves the myocardial contractile function (53, 54). These results confirm that IL-11 protects against CAD through the STAT3 pathway (54).

In contrast, current research has reported that high IL-11 expression may increase the risk of developing restenosis after stent implantation. In a model of carotid artery injury associated with plaque formation, compared with the control group, X203 injection significantly reduced the thickness of the vascular wall and plaque formation and suppressed the proliferation of VSMCs and collagen secretion. This result showed that inhibition of IL-11 decreases vascular fibrosis and reduces the risk of developing restenosis after vascular reconstruction (31). One possible explanation for these contradicting findings is that recombinant mouse IL-11 (rmIL-11) was primarily used to study rat and mouse disease models in more recent research, whereas rhIL-11, a competitive inhibitor of IL-11, was widely researched in rat or mouse cells in previous research (31). Therefore, Inhibition of IL-11 positively affected the rehabilitation of myocardial infarction and postoperative complications. Nevertheless, the changes and specific regulatory mechanisms of IL-11 in CAD progression remain to be elucidated.

### 4.2. Hypertension

Hypertension is a long-term increase in blood pressure to 140/90 mmHg or more, and it is an important risk factor for ischemic heart disease, stroke, and other cardiovascular diseases. According to conventional insights, the imbalance of the renin-angiotensin-aldosterone system and the sympathetic nervous system is the primary mechanism of hypertension development. Recent studies have concluded that immune cells also participate in the development of hypertension. Various immune cell may aggravate chronic inflammatory reactions in blood vessels, heart, kidney and brain, thereby destroying the blood pressure regulating function of these organs, and eliciting blood pressure to rise (55).

In a phase II clinical trial of using rhIL-11 in maging mild von Willebrand disease, it was found that the increase of IL-11 dose, 1 of 3 subjects had unstable hypertension at baseline (56). Another study showed that the average blood pressure of 24 patients with thrombocytopenic leukemia increased by 10 mmHg after receiving rhIL-11 treatment (57). This evidence suggests that IL-11 promotes the occurrence of hypertension.

To investigate how IL-11 specifically affects the occurrence of hypertension, Guo et al. established an Ang Ⅱ induced hypertension-related vascular model and found that the level of IL-11 in vascular adventitia increased. Injection of rmIL-11 aggravates Ang Ⅱ-mediated adventitia fibrosis. Conversely, IL-11 knockdown or injection with IL-11 neutralizing antibody downregulated adventitia fibrosis, macrophage infiltration, and the expression of inflammatory factors in mice. Moreover, *in vitro* experiments confirmed that IL-11 influences collagen production in the adventitia fibroblasts of thoracic aorta of rabbit through the non-classical ERK pathway (32). This result provides strong evidence for the role of IL-11 in hypertension, indicating that IL-11 promotes the development of hypertension mainly by promoting vascular adventitia remodeling.

Evidence has shown that IL-11 promotes pregnancy-induced hypertension in preeclampsia (PE). The IL-11 level is high in the serum of mouse with PE. The systolic blood pressure of pregnant rats treated with rhIL-11 in higher than that of the control group but withdrawing rhIL-11 reverses this phenomenon. These findings point to a potential role of IL-11 in regulating blood pressure during pregnancy by targeting pregnancy-associated plasma protein A2 (58).

### 4.3. Pulmonary hypertension

Pulmonary hypertension (PH), a chronic fatal disease, is characterized by high pulmonary arteriole resistance. Multiple molecular pathways work together to drive vasoconstriction, pulmonary artery remodeling, and progressive increase of vascular resistance, which eventually progress to right heart failure. Pulmonary vascular remodeling, a typical pathological feature of PH, is caused by the proliferation and migration of vascular wall cells, infiltration of inflammatory cells, and changes in the extracellular matrix (59).

In a clinical trial, it was found that the expression of IL-11 and IL-11RA in the pulmonary artery, pulmonary parenchyma, serum, and alveolar lavage fluid of patients with idiopathic pulmonary fibrosis (IPF) and PH was higher compared with the control group, and was prolonged than in patients with IPF but without PH. Immunofluorescence of pulmonary arteries revealed that the expression of IL-11 and IL-11RA is higher in patients with IPF and PH than in those with IPF alone (19). Another study revealed that IL-11 drives pulmonary vascular remodeling by inducing phenotype transformation of vascular wall cells. The pulmonary fibroblasts extracted from IL-11-treated mice were of endothelial origin, which suggests that IL-11 potentially activates the transformation of endothelial cells into the stroma. Moreover, IL-11 induced isolated pulmonary artery smooth muscle cells and human pulmonary artery microvascular endothelial cells to promote myofibroblast phenotype, which implies that IL-11 elicits phenotype transformation of vascular wall cells (19). The production of MMP-2 and MMP-9 in pulmonary arteries may be boosted by myofibroblast, which can also promote the deposition and cross-linking of collagen, leading to pulmonary vascular remodeling (60).

The JAK/STAT3 pathway, a key downstream pathway of IL-11, promotes pulmonary vascular remodeling. In a previous study, JAK2 and STAT3 expression in the pulmonary arteries of healthy individuals was not detected. Conversely, the expressions of JAK2 and STAT3 in the intima and media of pulmonary arterioles were high in the lung tissue sections of patients with IPF and PH (61). This result points to endothelial and smooth muscle cells of pulmonary arterioles as the primary cells for IL-11 production. Further evidence revealed that activation of STAT3 increases the expression of Pim1 (a proto-oncogene serine/threonine protein kinase) and nuclear factor of activated T cells 2, thus promoting smooth muscle cell proliferation and neointima formation (61). In the monocrotaline-induced PH rat model and chronic hypoxia-evoked PH mouse model, the clinically approved JAK1/JAK2 inhibitor ruxolitinib alleviates pulmonary vascular remodeling and reduces pulmonary artery pressure (61). IL-11 promotes pulmonary artery remodeling via the JAK/STAT3 pathway. However, the pathogenic factors of PH are complex, and further investigation is needed to determine the role of IL-11 in PH.

### 4.4. Cerebrovascular diseases

Cerebrovascular diseases are pathological diseases with impaired cerebral blood flow, including ischemic stroke, intracranial hemorrhage, and subarachnoid hemorrhage (62). Fang et al. found that IL-11 was elevated in the plasma of patients with spontaneous intracerebral hemorrhage, which was associated with hydrocephalus and mortality after intracerebral hemorrhage (63). Similarly, the serum IL-11 levels in patients with hypertensive intracerebral hemorrhage (HICH) were higher than those in the control group, reaching the peak on the 7th day after the onset. The serum IL-11 levels were lower than the baseline on the 14th day. The high expression of the serum IL-11 in HICH patients during treatment was associated with the high neurological deficit at discharge (64). These results indicate that but, the disease's severity, and the patient's prognosis.

Conversely, another research revealed that IL-11 mRNA and protein expression reduce considerably after cerebral ischemia. Treatment with rmIL-11 (20 g/kg) potentially minimizes cerebral infarction by suppressing apoptosis and proinflammatory cytokine expression following cerebral ischemia (65). In cases of cerebral bleeding and cerebral ischemia, the expression and effect of IL-11 are opposed. More studies are needed to determine the precise role of IL-11 in cerebrovascular disorders and its mechanisms.

### 4.5. Aortic dissection

Aortic dissection (AD) is a fatal vascular disease caused by aortic intima rupture or aortic wall bleeding, which leads to the separation of different aortic wall layers. The factors of its occurrence and development are generally classified into two categories; genetic and cytological factors. Genetic factors primarily include Marfan syndrome and familial thoracic aortic aneurysm (66). Cytological factors are related to the phenotypic transformation of aortic vascular smooth muscle cells and changes in extracellular matrix components. Vascular smooth muscle changes from a high contractile and low migratory contractile phenotype to a synthetic phenotype with reduced contractile capacity and increased mobility (67).

In a previous study, the level of IL-11 in plasma and thoracic aorta (especially in the torn segment of aortic dissection) of patients with acute thoracic aortic dissection increased. The aortic macrophages were one of the primary sources of IL-11 (68). Consequently, in a Marfan syndrome (MFS) model, the mRNA and protein expression of IL-11 was overexpressed in the aorta (especially in the aortic root and ascending aorta), and IL-11RA were overexpressed on aortic media VSMCs and adventitial fibroblasts (24).

In animal model studies involving overexpression or inhibition of IL-11, IL-11 is involved in the development of aortic dissection by inducing phenotype transformation of vascular smooth muscle and aortic vascular remodeling. To verify the influence of IL-11 on AD cytological factors, one study found that IL-11 overexpression in mice increased aortic remodeling, matrix, and inflammatory gene (such as LGALS3 and LAMP2) expression. This result suggests that IL-11 secreted by VSMCs causes aortic remodeling, fibrosis, and inflammation. Additional analysis revealed that X203 reduces the remodeling of the mouse aorta in two kinds of arterial pressure load models induced by aortic coarctation and Ang Ⅱ (42).

To investigate how IL-11 impacts the genetic factors of AD, another study established an MFS model for mice that were treated with the knockout of IL-11RA or X209 (IL-11RA antibody). Aortic fibrosis and inflammation were less severe, as well as the expression of fibrosis-related genes in the aorta. This result meant that inhibition of IL-11 is potentially beneficial to aortic atherosclerosis, aortic aneurysm, and aortic dissection. In-depth analysis revealed that the ERK pathway activates vascular smooth muscle in MFS mice, which is reversed by the knockout of the IL-11RA gene or X209 administration. This result implies that IL-11 potentially stimulates the phenotype transformation of VSMCs in an ERK-dependent manner. These studies further highlight the role of IL-11 in promoting aortic remodeling and the risk of developing aortic dissection, providing a basis for anti-IL-11 treatment to reduce the risk of developing aortic dissection (24).

### 4.6. Others

IL-11 is involved in angiogenesis in various tissues. In breast cancer, gastric cancer, colon cancer, and other malignant tumors, IL-11 promotes and accelerates angiogenesis in cancerous tissues (69–71). In patients with proliferative diabetic retinopathy (PDR), the expression of IL-11 and IL-11RA in pathological neovascular endothelial cells, leukocytes, and myofibroblasts of PDR preretinal vascular fibromembranes significantly increase. Meanwhile, the microvessel density and the number of PDR epiretinal membranes substantially and strongly correlate with the expression of IL-11 and IL-11RA in stromal cells (20). Other studies have shown that in rheumatoid arthritis (RA), IL-11 combined with IL-11RA in the endothelial cell directly activates RA angiogenesis. Moreover, IL-11 boosts fibroblasts to migrate and infiltrate into RA joints and secrete IL-8 and VEGF to indirectly promote RA angiogenesis (18).

In ischemic diseases, the administration of IL-11 improves blood supply. In mice with femoral artery ligation, pretreatment with rhIL-11 for 72 h before ligation increases the number of circulating CD34^+^/VEGFR2^+^ monocytes (which is known to support the growth of existing collateral vessels to reconstruct occluded vessels), which ultimately promotes the development of collateral vessels and increases perfusion recovery after femoral artery ligation (72). Although adipose-derived mesenchymal stem cells (ADSCs) promote angiogenesis in ischemic tissues, their poor viability after transplantation limits their clinical application. Studies have demonstrated that IL-11 enhances ADSCs' proliferation and migration and promotes cell survival and apoptosis resistance through the STAT3 signaling pathway. Blood perfusion in mice with ischemic limbs is enhanced by IL-11 overexpressed ADSCs *in vivo* (73). These results indicate that IL-11 has potential in the treatment of ischemic diseases. IL-11 is implicated in vasculitis. In an epigenomic analysis of CD14^+^ mononuclear cells from giant cell arteritis patients, a previous study found increased responsiveness of DNA differential methylation regions in the IL-11 pathway in patients in the active stage compared to the control group and patients in the remission stage. This result points to a potential involvement of IL-11 in giant cell arteritis (74).

## 5. Conclusion

In endothelial cells, IL-11 primarily promotes angiogenesis, thus ameliorating ischemic disease. IL-11 also promotes vascular remodeling by inducing phenotypic conversion of VSMCs and activation of fibroblasts, leading to corresponding target organ damage. In distinct vascular diseases, IL-11 works in various ways. IL-11 may be involved in the occurrence and progression of PH through the JAK/STAT3 pathway and in the onset of hypertensive aortic dissection via the non-classical ERK pathway. The inhibition of IL-11 signal can reduce vascular remodeling. Therefore, the study of drugs blocking IL-11 signal will have great clinical application value. Currently, little is known about the biological properties of IL-11. Further research is required to ascertain the function and precise mechanism of IL-11 in the development and progression of various vascular diseases, so as to provide targeted therapeutic drugs targeting IL-11 and help a large number of patients with vascular diseases worldwide.
